# Estrogen improves sevoflurane-induced cognitive dysfunction by regulating synaptic zinc homeostasis

**DOI:** 10.1186/s10020-025-01364-6

**Published:** 2025-10-14

**Authors:** Feixiang Li, Bingqing Gong, Tao Yang, Siwen Long, Jinqin Zhang, Yi Jiang, Yonghao Yu, Yongyan Yang, Dujuan Li

**Affiliations:** 1 Department of Anesthesiology, Tianjin Medical University General Hospital, Tianjin Institute of Anesthesiology, NO.154, Anshan Road, Heping District, Tianjin, 300052 China; 2 Department of Rehabilitation Medicine, The Second Affiliated Hospital of Wannan Medical College, No. 10 Kangfu Road, Wuhu, China; 3 Department of Anesthesiology, Tianjin Union Medical Center, the First Affiliated Hospital of Nankai University, Tianjin, China

**Keywords:** Cognition, Sevoflurane, Estrogen, Synaptic zinc, Znt3

## Abstract

**Background:**

Sevoflurane is known to induce cognitive dysfunction, but the underlying mechanisms remain unclear. Recent evidence suggests that disruptions in synaptic zinc homeostasis may contribute to neurotoxicity and cognitive impairment. This study investigates the role of synaptic zinc imbalance in sevoflurane-induced cognitive dysfunction and evaluates the neuroprotective effects of estrogen.

**Methods:**

Aged female C57BL/6 mice were exposed to sevoflurane to induce neurotoxicity. Synaptic zinc levels, Tau phosphorylation, synaptic vesicle numbers, neuronal firing frequency, and neuronal damage were assessed. The effects of zinc chelation with CaEDTA and estrogen supplementation on these parameters, as well as cognitive performance in the Morris water maze and Y-maze tests, were evaluated.

**Results:**

Sevoflurane exposure disrupts synaptic zinc homeostasis by upregulating Znt3 expression, leading to increased Tau phosphorylation, reduced synaptic vesicle numbers, decreased neuronal firing frequency, elevated neuronal death, and cognitive impairment. Chelation of zinc with CaEDTA attenuated Tau phosphorylation and neuronal death, enhanced neuronal firing, and improved cognitive function. Estrogen supplementation alleviates synaptic zinc imbalance by downregulating Znt3 expression, thereby reducing Tau phosphorylation and neuronal loss, increasing synaptic vesicle density and neuronal firing frequency, and improving cognitive function.

**Conclusions:**

This study reveals that sevoflurane-induced cognitive dysfunction is closely associated with synaptic zinc imbalance. Estrogen exerts its neuroprotective effects by restoring synaptic zinc homeostasis. These findings provide insights into the pathophysiological mechanisms underlying anesthesia-related cognitive impairment and highlight the therapeutic potential of estrogen in perioperative neuroprotection.

**Graphical Abstract:**

Estrogen supplementation can mitigate sevoflurane-induced cognitive impairment by restoring synaptic zinc homeostasis.

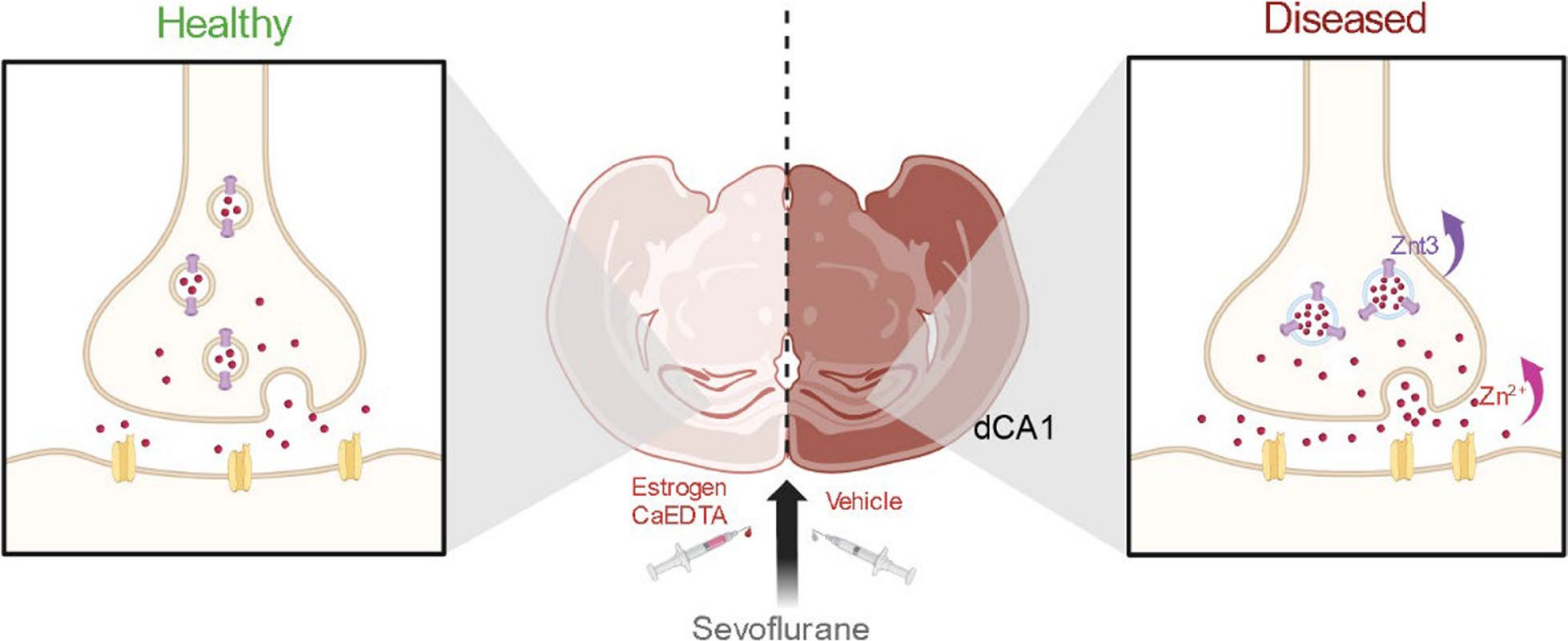

**Supplementary Information:**

The online version contains supplementary material available at 10.1186/s10020-025-01364-6.

## Introduction

Cognitive dysfunction is a common complication associated with various neurodegenerative diseases and perioperative anesthesia, significantly affecting patients’ quality of life and postoperative recovery [1]. In recent years, perioperative cognitive dysfunction has emerged as a research focus, particularly regarding the potential role of general anesthetics in its development. Sevoflurane, a widely used volatile anesthetic, is known for its excellent anesthetic properties and controllability but has also been shown to induce both short-term and long-term damage to the central nervous system [2, 3]. Substantial evidence suggests that sevoflurane impairs neuronal function through multiple pathways, leading to cognitive decline [4–6]. However, its precise mechanisms remain incompletely understood and warrant further investigation.

The adverse effects of sevoflurane on synaptic function, a key aspect of its neurotoxicity, have been well-documented [7, 8]. Synaptic zinc, an important neuroregulatory factor, plays a critical role in the central nervous system, particularly in regions such as the hippocampus and cerebral cortex, which are closely associated with learning and memory [9, 10]. Under normal conditions, synaptic zinc maintains homeostasis through the dynamic regulation of specific zinc transporters, crucial for synaptic transmission, plasticity, and memory formation [11]. Zinc transporter 3 (Znt3), a member of the SLC30A family, is responsible for transporting zinc ions into synaptic vesicles and releasing them into the synaptic cleft during neurotransmitter release [12]. This process is vital for maintaining synaptic zinc homeostasis, and any disruption in this balance can lead to excitotoxicity, synaptic structural damage, and functional impairment, ultimately resulting in cognitive deficits and other neuropsychiatric disorders [13, 14]. Based on these insights, we hypothesize that dysregulation of synaptic zinc may be a key mechanism underlying sevoflurane-induced neurotoxicity.

Estrogen, a hormone with a wide range of biological functions, has recently garnered significant attention for its neuroprotective effects. Studies have shown that estrogen can regulate neuronal survival, synapse formation, and functional recovery through its receptor pathways [15]. In terms of synaptic zinc homeostasis, estrogen helps maintain the dynamic balance of zinc by acting on zinc transport-related proteins, thereby protecting neurons from toxic damage caused by excess free zinc [16]. Estrogen replacement therapy can influence the serotonergic system by regulating the expression and function of serotonin transporters and receptors, thereby improving anxiety and depression. It has also been shown to decrease the risk and severity of Alzheimer’s disease in postmenopausal women, potentially through suppression of Aβ production [17, 18]. However, the specific role of estrogen in sevoflurane-induced cognitive impairment remains unclear.

Based on the above background, this study hypothesizes that sevoflurane induces central nervous system damage and cognitive dysfunction by disrupting synaptic zinc homeostasis, and that estrogen supplementation may exert a neuroprotective effect by regulating synaptic zinc balance, thereby alleviating cognitive dysfunction. By exploring the interaction between sevoflurane and estrogen in the regulation of synaptic zinc homeostasis and cognitive function, this study aims to provide a novel pathophysiological explanation for anesthesia-related cognitive dysfunction and open new avenues for the prevention and treatment of perioperative cognitive disorders.

## Materials and methods

### Animals

**Fig. 1:**
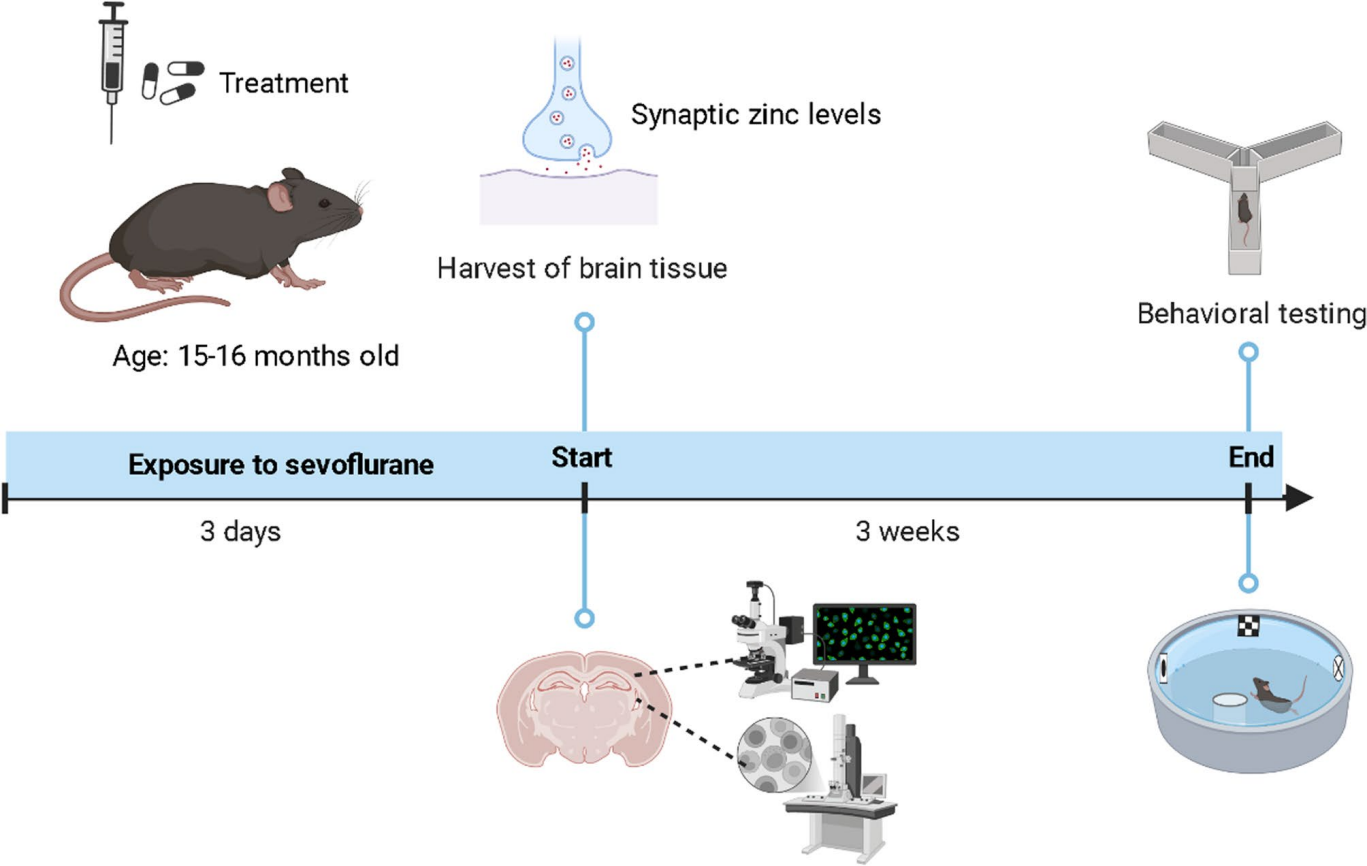
Timeline flowchart of the experimental design

We used female C57BL/6 mice (15–16 months old, weighing 20–25 g) and acclimated the animals for one week before the experiment. They were housed under a 12-hour light/dark cycle, with an ambient temperature of 22–24 °C and humidity of 50–60%. All experiments, except for the behavioral ones, were conducted with 6 mice per group, while the behavioral experiments involved 10 mice per group. All experimental procedures were conducted in accordance with the “Regulations for the Administration of Laboratory Animals” and approved by the Animal Ethics Committee. The schematic timeline of the experimental design is shown in Fig. 1.

### Sevoflurane exposure and Estrogen treatment

The sevoflurane exposure protocol was conducted as previously described [3]. Briefly, mice were exposed to 3% sevoflurane in 60% oxygen for 2 h per day over three consecutive days, while control mice were exposed to 60% oxygen alone. During the exposure, rectal temperature was maintained at 37 °C. Estrogen treatment (0.2 mg/kg) was administered subcutaneously 1 h prior to each sevoflurane exposure, based on dosages used in earlier studies [19].

### Stereotactic injection, caedta, and AAV treatment

The procedure for stereotactic injection followed the previously described protocol [20]. Briefly, after isoflurane anesthesia, the mice were secured in a stereotaxic apparatus, and the skull was leveled. Following scalp sterilization and a midline incision, a small burr hole was drilled at the target site (AP-1.1 mm× ML ± 1.5 mm, 0.5 mm × 0.5 mm). A micropipette preloaded with CaEDTA (200 nmol, 2 µL), adeno-associated virus overexpressing Znt3 (AAV-Znt3, 500 nL), or an equal volume of control vector was inserted to the target depth (2.5 mm). The solution was injected slowly, and the needle was kept in place for 5 min to minimize backflow before being withdrawn gradually. The incision was sutured, and the mice were monitored in a heated recovery chamber. Once fully awake, the mice were returned to their cages. CaEDTA treatment was administered 30 min prior to sevoflurane exposure. After the injection, mice were left undisturbed for the designated period before undergoing the sevoflurane exposure protocol. For AAV-injected mice, experiments were performed two weeks post-injection to allow for adequate viral expression.

### Western blotting

Western blotting (WB) was carried out according to protocols reported in prior research [21]. Briefly, after the experiment, mice were sacrificed, and hippocampal tissue was extracted. The tissue was lysed using a lysis buffer containing protease and phosphatase inhibitors, and the supernatant was collected to determine the total protein concentration. Equal amounts of protein were mixed with sample buffer containing DTT, boiled at 100 °C, and then separated by SDS-PAGE. After electrophoresis, proteins were transferred onto a PVDF membrane using a semi-dry transfer technique. The membrane was then blocked with 10% non-fat milk for 1.5 h and incubated overnight at 4 °C with primary antibodies targeting the desired proteins (AT8, 1:1500, Thermo Scientific, USA; Znt3, 1:1200, Proteintech, China; Ap3δ, 1:1000, Affinity, USA). Following incubation, the membrane was rinsed with TBST buffer and treated with HRP-conjugated secondary antibodies. After washing, chemiluminescent signals were detected using the ECL system, and images were recorded using an imaging system. Finally, the protein bands were quantified by measuring the optical density using ImageJ software, and the data were normalized to internal controls (GAPDH, 1:5000, Affinity, USA). Group differences were analyzed using appropriate statistical methods.

### Immunofluorescence

First, mice were sacrificed, and brain tissue was extracted and fixed in 4% paraformaldehyde solution at room temperature for 1 h. The tissues were then washed three times with PBS buffer to remove excess fixative. The tissue was sectioned into 10 μm thick slices and permeabilized at room temperature for 1 h with 0.3% Triton X-100 solution to enhance antibody penetration. After permeabilization, the slices were blocked with 5% BSA for 2 h at room temperature, and then incubated with diluted primary antibody solution (Znt3, 1:100, Proteintech, China) overnight at 4 °C. The next day, the slices were rinsed three times with PBS, each wash lasting 5 min, and then incubated with fluorescently labeled secondary antibody solution for 2 h at room temperature. After incubation, the slices were washed again and stained with DAPI for 30 min to label the cell nuclei. Finally, the slices were mounted with a mounting medium, and fluorescence signals were observed under a fluorescence microscope. Images were captured and analyzed.

### Transmission electron microscopy

After anesthetizing the mice and performing perfusion, the hippocampal tissues were extracted and soaked in electron microscopy fixative. Subsequently, the tissues were post-fixed with 1% osmium tetroxide to enhance contrast. The samples were dehydrated through a graded ethanol series and embedded in epoxy resin to form hardened blocks. Ultrathin Sect. (70 nm) were then cut using an ultramicrotome and placed on nickel grids. The sections were treated with uranyl acetate and lead citrate for staining, then examined under a transmission electron microscope to capture images.

### TUNEL staining

After the experiment, the mice were euthanized, and their brain tissues were collected and fixed in 10% neutral formalin solution at room temperature for 1 day. The tissues were then washed with water and embedded in paraffin. The tissue sections were cut to a thickness of 4–5 μm, followed by deparaffinization and rehydration. Deparaffinization was performed using a gradient ethanol solution, and the sections were washed with PBS. To remove the DNA fragment end protection, the sections were treated with DNase-free proteinase K for 20 min, followed by PBS washing. Next, the sections were stained using a TUNEL kit according to the manufacturer’s instructions. Specifically, the sections were incubated with terminal deoxynucleotidyl transferase (TdT) reaction mixture at 37 °C for 1 h. After staining, the sections were counterstained with DAPI to label the cell nuclei. Finally, the sections were examined using a fluorescence microscope, and images were acquired. Apoptotic cells were indicated by green fluorescence, and the apoptosis index (TUNEL+/NeuN + double-positive cells) was calculated for each group. All experimental data were analyzed quantitatively using ImageJ software.

### Morris water maze

Before the experiment, all animals were acclimated in the maze environment for one week. The Morris Water maze was composed of a circular pool with a diameter of 1.2 m, containing a hidden platform positioned 1 cm beneath the water’s surface. The water temperature was kept at 22 ± 2° C. Non-toxic white dye was used to make both the platform and the water appear white. Multiple visual cues were placed around the maze to aid in navigation. The experiment was structured into a training phase and a testing phase. The training phase spanned 5 days, with each trial starting from a different location in the maze. Animals were given 90 s to locate the platform, and if they failed to find it within the allotted time, they were gently guided to the platform and allowed a 15-second resting period. During the training phase, the latency for the mice to find the platform (the time taken to reach the platform from the entry point) was recorded. The testing phase took place on day 6, with the platform removed. The animals were allowed to swim for 90 s, and the time spent in the target quadrant as well as the number of platform crossings were recorded. Data were analyzed using a video tracking system. The latency to find the platform and the number of platform crossings were used as indicators of spatial memory.

### Y-maze

Before the experiment, the animals were acclimated to the environment for 72 h. The Y-maze consisted of three connected arms: the starting arm, the novel arm, and the familiar arm. The experiment was split into two parts: the training phase and the testing phase. In the training phase, mice were positioned in the starting arm and given 10 min to explore the familiar arms at their own pace. The testing phase was conducted one hour after the completion of the training phase. In the testing phase, mice were placed in the starting arm and allowed to choose the previously unexplored arm, with the exploration lasting for 5 min. Spatial memory ability was assessed by counting the number of entries into the novel arm and measuring the time spent exploring the novel arm during the testing phase.

### Open field test

The open field test was conducted to assess the locomotor activity of mice. Each mouse was individually placed in the center of a square open field arena (50 × 50 × 40 cm) with a white floor and enclosed walls. The animals were allowed to explore freely for 5 min. Their movements were recorded using an automated video tracking system.

The total distance traveled during the test period was measured as an index of locomotor activity. The apparatus was cleaned with 75% ethanol between trials to eliminate odor cues.

### Synaptic separation and synaptic zinc measurement

The synaptosome isolation procedure was performed as previously described [22]. Briefly, hippocampal tissues were rapidly collected from experimental animals postmortem and homogenized in ice-cold isotonic buffer. The homogenate was first centrifuged at low speed to remove nuclei and cellular debris, followed by medium-speed centrifugation to obtain crude synaptosomes. The resulting pellet was resuspended in ultrapure water to induce osmotic lysis and release synaptic vesicles. A second round of ultracentrifugation was then performed to pellet the synaptic vesicles. The synaptic vesicle fraction was further purified using a sucrose density gradient centrifugation to obtain highly purified preparations.

Synaptic vesicle zinc levels were assessed using a fluorescent zinc probe method. Synaptosome suspensions were incubated with a zinc-specific fluorescent probe at 37 °C for 45 min and then washed with PBS to remove unbound probes. Fluorescence intensity was measured using a fluorescence spectrophotometer at specific wavelengths. Zinc ion concentrations were quantified against a standard curve, and differences between experimental and control groups were analyzed.

### In vivo electrophysiology

Mice were anesthetized with isoflurane and placed in a stereotaxic frame. A small cranial window was created, and a tungsten microelectrode (16-channel microwire) was inserted into the hippocampal CA1 region. The electrode implantation was performed two weeks prior to the modeling procedure, and data collection was conducted three weeks after the completion of modeling. Following the experimental procedures, local neuronal firing activity was recorded under resting conditions. Signal amplification was achieved using a differential amplifier, and data were collected through a digital acquisition system.

### Statistical analysis

Statistical analyses were carried out using SPSS 26.0 or GraphPad Prism 8. Data are presented as the mean ± standard deviation (SD) or median (interquartile range, IQR) as appropriate. Normality of data distribution was assessed using the Shapiro–Wilk test. For comparisons between two groups, either an independent-samples t-test or the Mann–Whitney U test was performed depending on the distribution of the data. For comparisons involving multiple groups with two independent variables, a two-way analysis of variance (two-way ANOVA) was conducted to assess the main effects and interaction between factors, followed by Bonferroni-corrected pairwise comparisons. When the assumptions of normality or homogeneity of variance were not met, non-parametric methods were applied, including the Kruskal–Wallis test with Dunn’s multiple comparisons. Statistical differences are indicated as follows: * for *P* < 0.05, ** for *P* < 0.01, *** for *P* < 0.001, and ns for no significant difference.

## Results

### Sevoflurane disrupts synaptic zinc homeostasis, induces neuronal damage, and impairs cognitive function

**Fig. 2:**
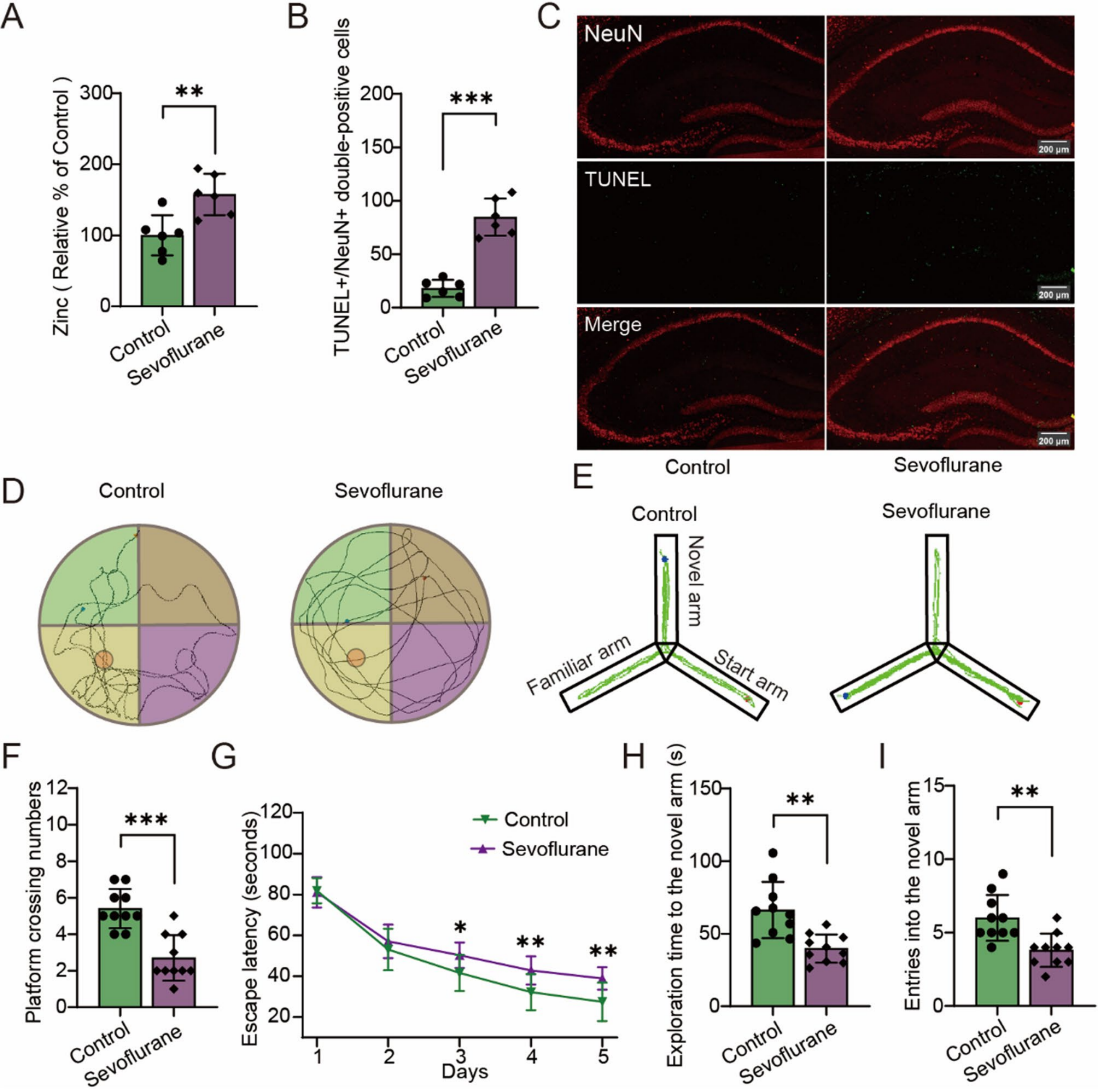
Neurotoxicity induced by sevoflurane. **A** Level of synaptic zinc (*n* = 6/group). **B** and **C** represent the number of TUNEL+/NeuN + double-positive cells and representative images in the hippocampus (*n* = 6/group). **D** and **E** illustrate path maps from the Morris Water maze and Y-maze, respectively. **F** and **G** show the frequency of platform crossings and the escape latency in the Morris water maze, respectively. (*n* = 10/group). **H** and **I** represent the number of entries into the novel arm and the time spent exploring the novel arm in the Y-maze, respectively (*n* = 10/group). Student’s t-test: (**A**), (**B**), (**F**) and (**H**). Two-way ANOVA: (**G**). *P < 0.05; **P < 0.01; ***P < 0.001

To evaluate the effect of sevoflurane on synaptic zinc, we measured synaptic zinc levels and observed a significant increase following sevoflurane exposure (Fig. 2A, t (10) = −3.465, *P* = 0.006). In addition, TUNEL staining revealed a marked increase in neuronal cell death in the hippocampus after sevoflurane exposure (Fig. 2B, C, t (10) = −8.551, *P* < 0.001). In behavioral assessments, the Morris Water maze showed that sevoflurane exposure significantly reduced the frequency of platform crossings and prolonged the latency to locate the hidden platform (Fig. 2D, F, G, t (18) = 5.175, *P* < 0.001; *F* (1,18) = 6.394, *P* = 0.021). Similarly, in the Y-maze, sevoflurane decreased the number of novel arm entries and the time spent exploring the novel arm (Fig. 2E, H, I, t (18) = 3.601, *P* = 0.002; *t* (18) = 3.874, *P* = 0.001). The open field test results indicated that sevoflurane exposure did not affect the locomotor activity of mice (Supplementary Fig. 1 A, t (18) = 0.169, *P* = 0.868).

### CaEDTA reduces neurotoxicity in mice exposed to Sevoflurane

**Fig. 3:**
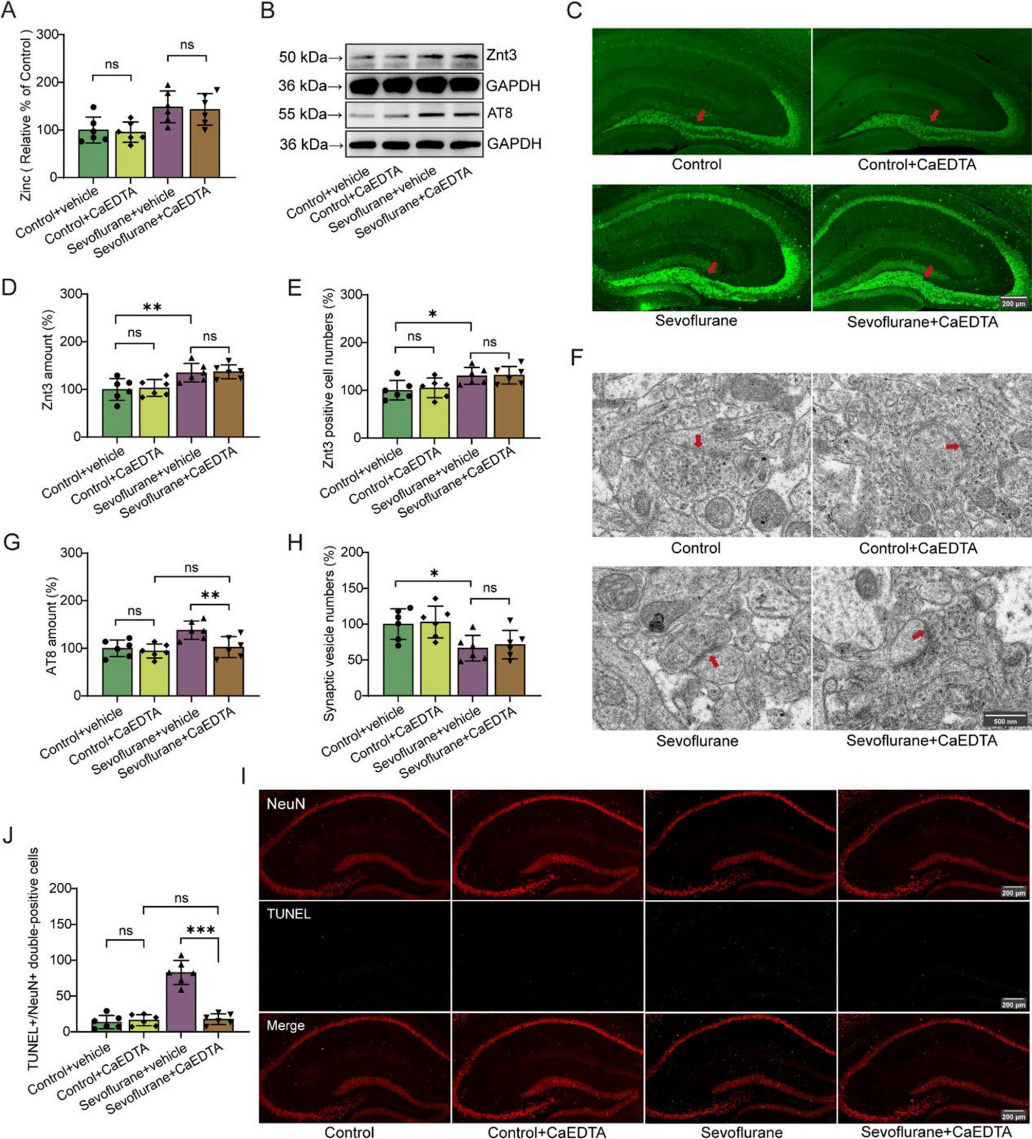
CaEDTA reduces neurotoxicity in mice exposed to sevoflurane. **A** Level of synaptic zinc (*n* = 6/group). **B**, **D**, **G** Quantification of Znt3 and AT8 proteins (*n* = 6/group). **C** and **E** represent the number of Znt3-Positive cells and representative images in the hippocampus (marked with red arrows, *n* = 6/group). **F** and **H** represent the number of synaptic vesicles and the representative transmission electron microscopy (TEM) images of synapses (marked with red arrows), respectively (*n* = 6/group). **I** and **J** represent the number of TUNEL+/NeuN + double-positive cells and representative images in the hippocampus (*n* = 6/group). Two-way ANOVA: (**A**), (**D**), (**E**), (**G**), (**H**) and (**J**). *P < 0.05; **P < 0.01; ***P < 0.001; ns, no significance

**Fig. 4:**
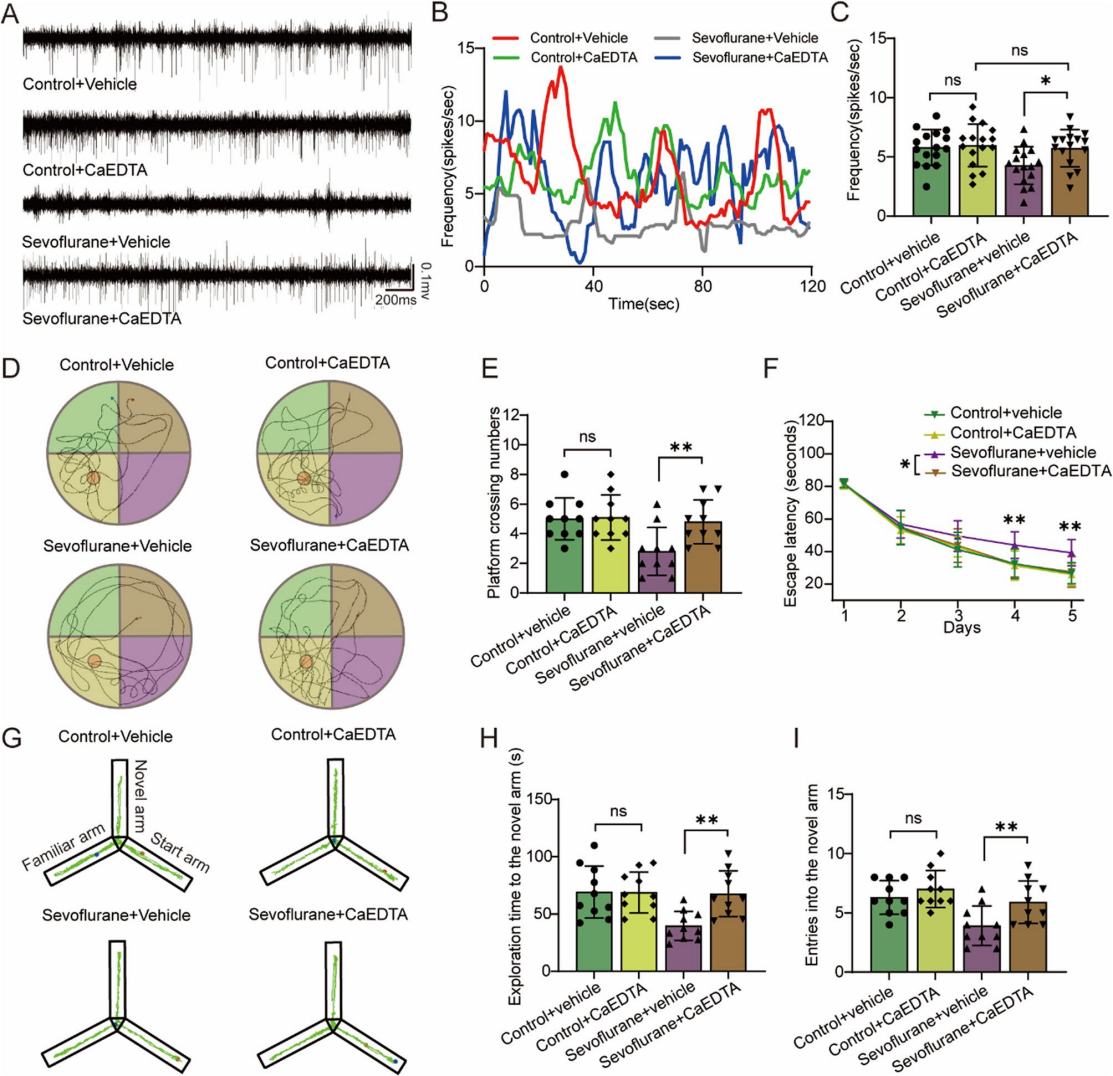
CaEDTA reversed the sevoflurane-induced decrease in neuronal firing frequency and cognitive dysfunction. **A**-**C** show the firing frequency and representative trajectories of hippocampal neuronal activity (*n* = 6/group). **D** and **G** illustrate path maps from the Morris Water maze and Y-maze, respectively. **E** and **F** show the frequency of platform crossings and the escape latency in the Morris water maze, respectively (*n* = 10/group). **H** and **I** represent the frequency of entries into the novel arm and the time spent exploring the novel arm in the Y-maze, respectively (*n* = 10/group). Two-way ANOVA: (**C**), (**F**), (**G**) (**H**) and (**I**). *P < 0.05; **P < 0.01; ns, no significance

To further explore the role of synaptic zinc in sevoflurane-induced neurotoxicity, we employed CaEDTA to neutralize zinc ions released from synaptic vesicles into the synaptic cleft. Notably, CaEDTA did not alter synaptic zinc levels in either the control or sevoflurane-exposed groups (Fig. 3A, F (1,20) = 0.175, *P* = 0.068). Western blotting and immunofluorescence analyses revealed that sevoflurane exposure increased Znt3 protein expression in the mouse hippocampus (F (1,20) = 10.203, *P* = 0.005; F (1,20) = 7.373, *P* = 0.013), whereas CaEDTA had no significant effect on Znt3 expression (Figure. 3B-E, F (1,20) = 0.11, *P* = 0.743; F (1,20) = 0.173, *P* = 0.682). Interestingly, while CaEDTA did not affect Tau phosphorylation (AT8) levels in the control group (*F* (1,20) = 0.284, *P* = 0.6), it obvious lowered these levels in the sevoflurane-exposed group (Fig. 3B, G, F (1,20) = 10.993, *P* = 0.003). We also found that sevoflurane exposure reduced the number of synaptic vesicles in the hippocampal CA1 region (*F* (1,20) = 8.154, *P* = 0.01), and this reduction was not alleviated by CaEDTA treatment (Fig. 3F, H, F (1,20) = 0.189, *P* = 0.669). Furthermore, TUNEL staining revealed that CaEDTA alleviated neuronal cell death in the hippocampus of sevoflurane-exposed groups (Fig. 3I, J, F (1,20) = 48.528, *P* < 0.001). In vivo electrophysiological recordings showed that CaEDTA did not affect the firing frequency of neurons in the hippocampal CA1 region of the control group (*F* (1,60) = 0.107, *P* = 0.744), However, it reversed the sevoflurane-induced reduction in neuronal firing frequency (Fig. 4A-C, F (1,60) = 6.445, *P* = 0.014).

### CaEDTA mitigated the cognitive dysfunction induced by Sevoflurane

In the Morris water maze test, we observed that CaEDTA had no effect on the performance of control mice, but significantly increased the frequency of platform crossings and reduced the latency to locate the hidden platform in sevoflurane-exposed mice (Fig. 4D-F, F (1,18) = 4.917, *P* = 0.039; *F* (1,36) = 8.77, *P* = 0.005). Similarly, in the Y-maze, CaEDTA had no effect on the control group but enhanced the frequency of entries into the novel arm and the duration spent exploring the novel arm in the sevoflurane-exposed group (Fig. 4G-I, F (1,36) = 7.668, *P* = 0.009; *F* = 11.292, *P* = 0.002).

### Estrogen improves synaptic zinc homeostasis and reduces neurotoxicity in mice exposed to Sevoflurane

**Fig. 5:**
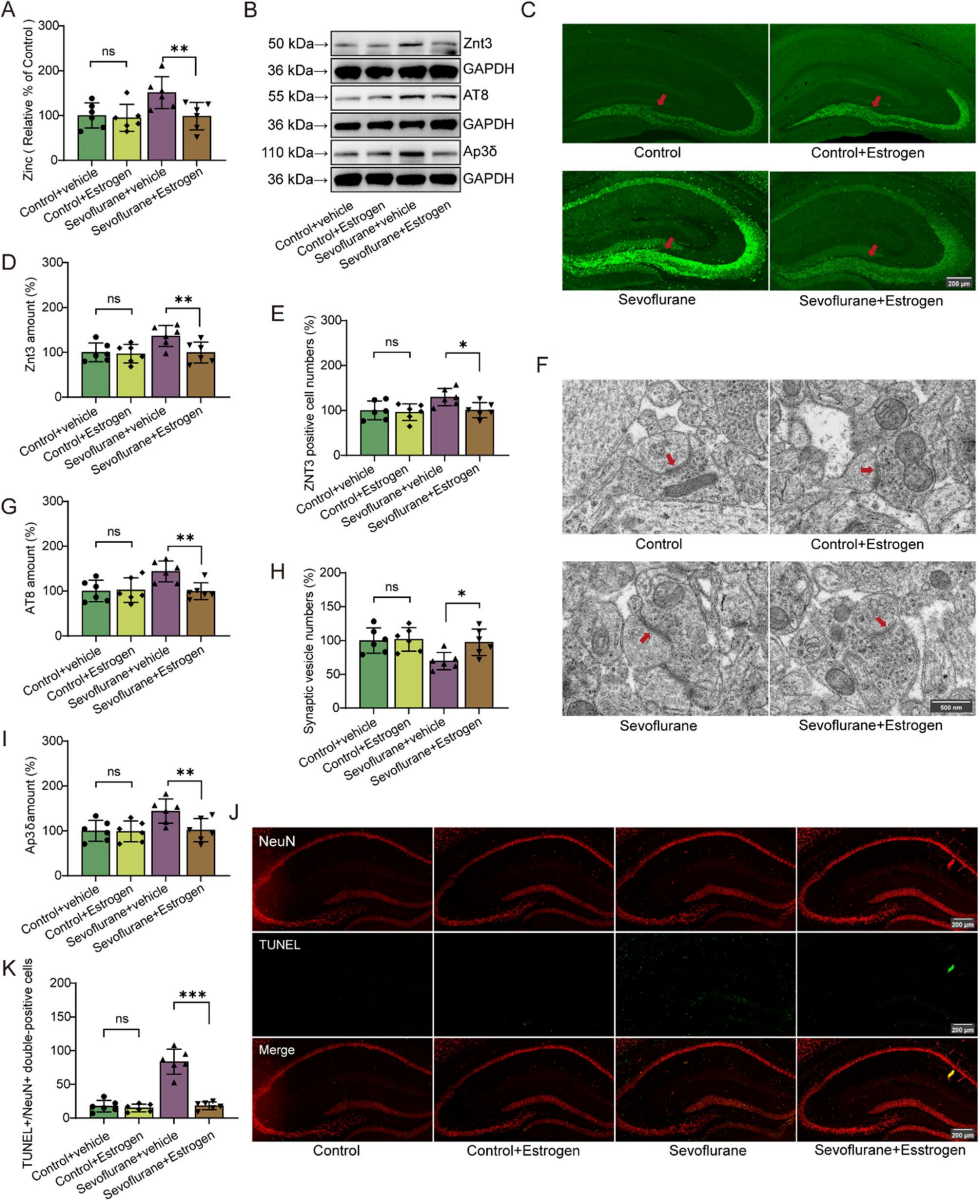
Estrogen improves synaptic zinc homeostasis and reduces neurotoxicity in mice exposed to sevoflurane. **A** Level of synaptic zinc (*n* = 6/group). **B**, **D**, **G**, **I** Quantification of Znt3, AT8 and Ap3δ (*n* = 6/group). **C** and **E** represent the number of Znt3-Positive cells and representative images in the hippocampus (marked with red arrows, *n* = 6/group). **F** and **H** represent the number of synaptic vesicles and the representative transmission electron microscopy (TEM) images of synapses (marked with red arrows), respectively (*n* = 6/group). **J** and **K** represent the number of TUNEL+/NeuN + double-positive cells and representative images in the hippocampus (*n* = 6/group). Two-way ANOVA: (A), (D), (E), (G), (H), (I) and (K). *P < 0.05; ***P < 0.001; ns, no significance

To evaluate the effect of estrogen supplementation, we measured changes in estrogen levels in the hippocampus, as this region is closely associated with cognitive function (Supplementary Fig. 1B, t (10) = −6.041, *P* < 0.001). To further investigate the effect of estrogen on sevoflurane-induced synaptic zinc imbalance, we analyzed synaptic zinc ion levels and found that estrogen reduced zinc ion levels in synaptic vesicles of the sevoflurane group (Fig. 5A, F (1,20) = 8.494, *P* = 0.009). Western blotting and immunofluorescence results showed that while estrogen did not affect Znt3 protein expression in the control group (*F* (1,20) = 0.058, *P* = 0.812; *F* (1,20) = 0.117, *P* = 0.736), it notably decreased Znt3 protein expression in the sevoflurane-exposed group (Fig. 5B-E, F (1,20) = 8.54, *P* = 0.008; *F* (1,20) = 7.104, *P* = 0.015). Additionally, estrogen lowered Tau phosphorylation (AT8) levels in the sevoflurane-exposed group (Fig. 5B, G, F (1,20) = 10.594, *P* = 0.004). Similarly, TUNEL staining revealed that estrogen attenuated neuronal cell death the hippocampus induced by sevoflurane (Fig. 5J, K, F (1,20) = 58.194, *P* < 0.001). We also found that estrogen mitigated the sevoflurane-induced reduction in the number of synaptic vesicles in the hippocampal CA1 region (Fig. 5F, H, F (1,20) = 7.715, *P* = 0.012). Given that Ap3δ significantly influences Znt3 expression, we further examined Ap3δ expression [23]. Western blotting analysis revealed that sevoflurane increased Ap3δ expression, whereas estrogen effectively reduced Ap3δ expression (Fig. 5B, I, F (1,20) = 8.711, *P* = 0.008).

**Fig. 6:**
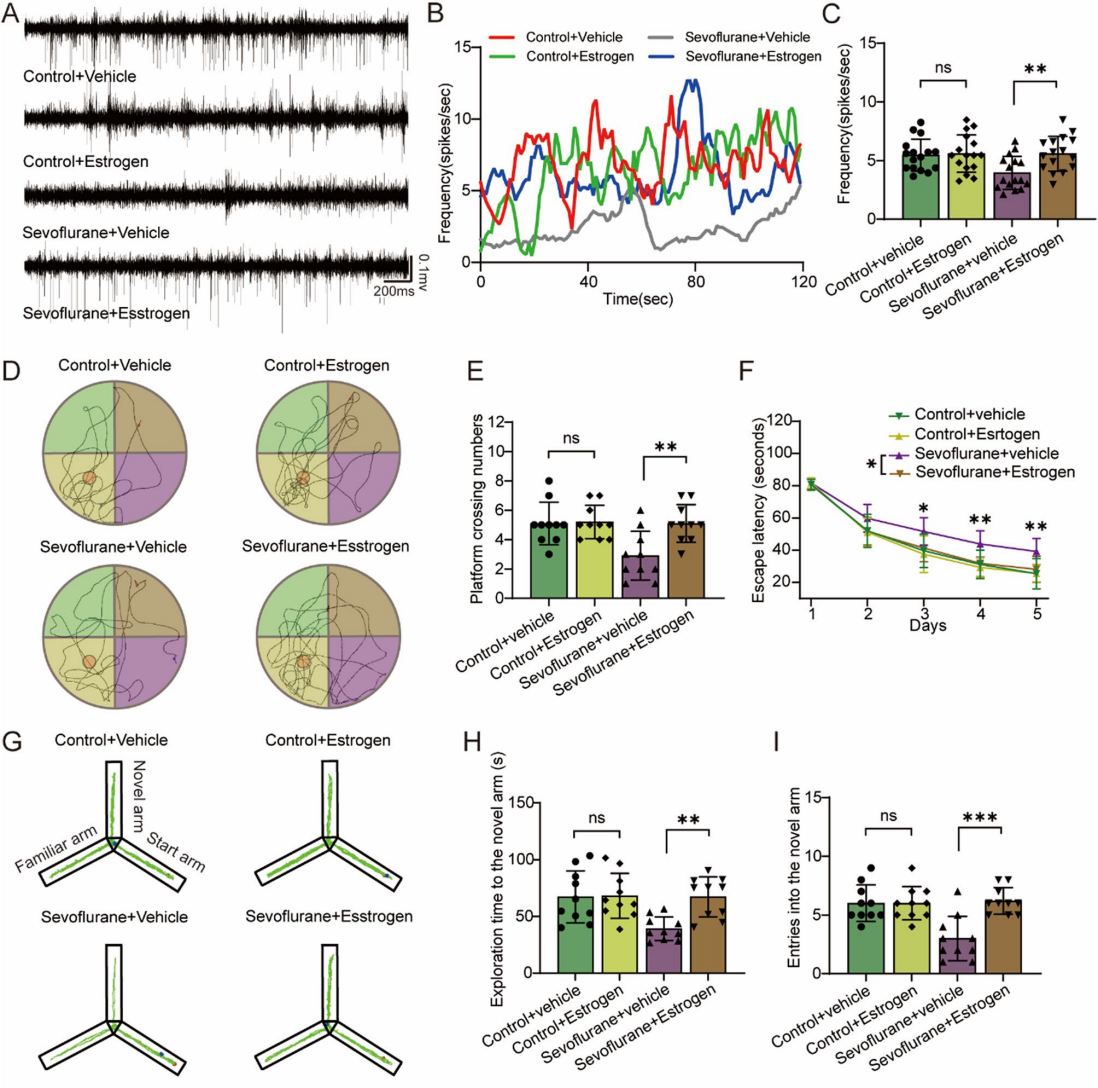
Estrogen reversed the sevoflurane-induced decrease in neuronal firing frequency and cognitive dysfunction. **A**-**C** show the firing frequency and representative trajectories of hippocampal neuronal activity (*n* = 6/group). **D** and **G** illustrate path maps from the Morris Water maze and Y-maze, respectively. **E** and **F** show the frequency of platform crossings and the escape latency in the Morris water maze, respectively (*n* = 10/group). **H** and **I** represent the frequency of entries into the novel arm and the time spent exploring the novel arm in the Y-maze, respectively (*n* = 10/group). Two-way ANOVA: (C), (F), (G) (H) and (I). *P < 0.05; **P < 0.01; ***P < 0.001; ns, no significance

In vivo electrophysiological recordings showed that estrogen did not affect the firing frequency of neurons in the hippocampal CA1 region of the control group (*F* (1,60) = 0.058, *P* = 0.81), However, it reversed the sevoflurane-induced reduction in neuronal firing frequency (Fig. 6A-C, F (1,60) = 9.972, *P* = 0.002).

### Estrogen alleviates sevoflurane-induced dysfunction

In the Morris water maze test, we observed that estrogen increased the frequency of platform crossings and reduced the latency to find the hidden platform in mice exposed to sevoflurane (Fig. 6D-F, F (1,18) = 7.674, *P* = 0.013; *F* (1,36) = 12.393, *P* = 0.001). In the same way, in the Y-maze, estrogen enhanced the number of entries into the novel arm and the time spent exploring it in the sevoflurane-exposed group (Fig. 6G-I, F (1,36) = 22.048, *P* < 0.001; *F* (1,36) = 11.778, *P* = 0.002).

### AAV-Znt3 abolished the neuroprotective effects of Estrogen

**Fig. 7:**
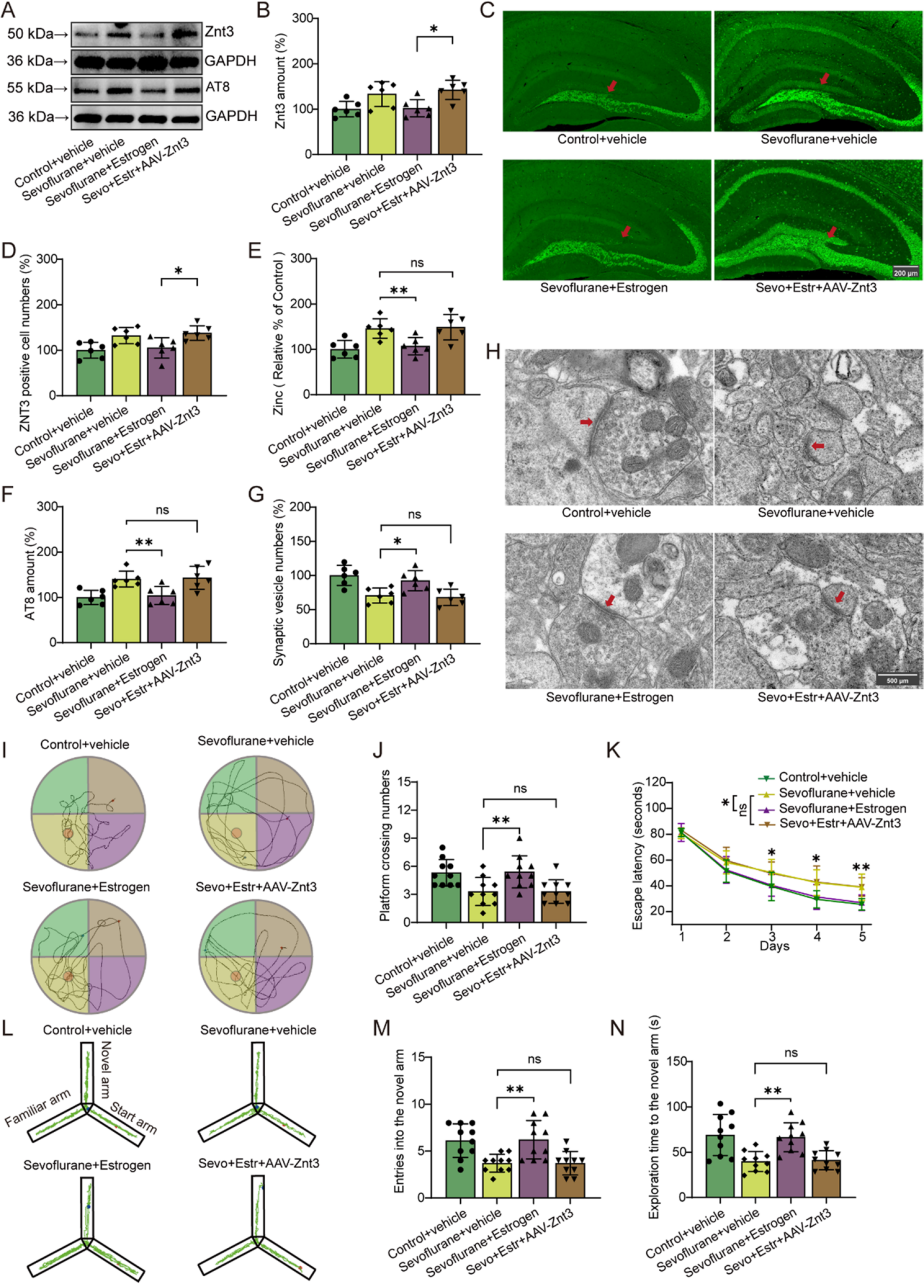
Effect of AAV-Znt3 on the neuroprotective role of estrogen. **A**, **B**, **G** Quantification of Znt3 and AT8 proteins (*n* = 6/group). **C** and **D** represent the number of Znt3-Positive cells and representative images in the hippocampus (marked with red arrows, *n* = 6/group). **E** Level of synaptic zinc (*n* = 6/group). **G** and **H** represent the number of synaptic vesicles and the representative transmission electron microscopy (TEM) images of synapses (marked with red arrows), respectively (*n* = 6/group). (I) and (L) illustrate path maps from the Morris Water maze and Y-maze, respectively. **J** and **K** show the frequency of platform crossings and the escape latency in the Morris water maze, respectively (*n* = 10/group). **M** and **N** represent the frequency of entries into the novel arm and the time spent exploring the novel arm in the Y-maze, respectively (*n* = 10/group). Two-way ANOVA: (B), (D), (E), (F), (G), (J), (K), (M) and (N). *P < 0.05; **P < 0.01; ns, no significance. Sevo, Sevoflurane; Estr, Estrogen

To explore the role of Znt3 in synaptic zinc homeostasis and estrogen-mediated neuroprotection, we overexpressed Znt3 using an adeno-associated virus. We first validated the effect of AAV-Znt3, and the results are shown in Supplementary Fig. 1 C (*t* (10) = −2.871, *P* = 0.017). Western blot and immunofluorescence confirmed that AAV-Znt3 significantly increased hippocampal Znt3 expression in sevoflurane-exposed mice treated with estrogen (Fig. 7A–D; *P* = 0.024, *P* = 0.040). Estrogen treatment reduced synaptic vesicular zinc levels in sevoflurane-exposed mice, but this effect was abolished by Znt3 overexpression (Fig. 7E; *P* = 0.007 vs. *P* = 0.830).

We found that estrogen significantly decreased Tau phosphorylation at AT8 sites in the hippocampus, and this reduction was blocked by AAV-Znt3 (Fig. 7A, F; *P* = 0.005 vs. *P* = 0.804). Estrogen also reversed the sevoflurane-induced reduction in synaptic vesicle density in the CA1 region, an effect similarly blocked by Znt3 overexpression (Fig. 7G, H; *P* = 0.010 vs. *P* = 0.741).

In the Morris water maze test, estrogen increased the number of platform crossings and reduced the latency to locate the hidden platform in sevoflurane-exposed mice (Fig. 7I, J, K, *P* = 0.003; *F* (1,18) = 8.37, *P* = 0.01); however, these beneficial effects were blocked by AAV-Znt3 (*P* = 1; *F* (1,18) = 0.082, *P* = 0.778). Similarly, in the Y-maze test, estrogen enhanced both the number of entries into the novel arm and the time spent exploring it (Fig. 7L, M, N, *P* = 0.001; *P* = 0.001), but these effects were also abolished by AAV-Znt3 (*P* = 1; *P* = 0.839).

## Discussion

This study investigated the underlying mechanism by which sevoflurane induces cognitive dysfunction through disruption of synaptic zinc homeostasis and evaluated the therapeutic effects of estrogen supplementation in mitigating these impairments. The results demonstrated that sevoflurane significantly disrupted synaptic zinc homeostasis by upregulating Znt3 expression, leading to a reduction in synaptic vesicles, decreased neuronal firing frequency, neuronal damage, and cognitive impairment. Estrogen supplementation downregulated Znt3 expression and effectively alleviated these effects by restoring synaptic zinc balance. These findings offer new insights into the pathophysiology of anesthesia-related cognitive dysfunction and provide a theoretical basis for the development of targeted therapeutic strategies.

Firstly, this study further corroborates the critical role of synaptic zinc homeostasis in cognitive function. As a key neuromodulatory factor, synaptic zinc is transported into synaptic vesicles by Znt3 and released into the synaptic cleft during neurotransmitter exocytosis, which is essential for maintaining synaptic transmission, neuronal plasticity, and memory processes [24]. However, excessive accumulation of free zinc can induce neurotoxicity through mechanisms such as oxidative stress, inflammatory responses, and apoptosis [25]. In this study, sevoflurane exposure increased Znt3 expression, disrupting synaptic zinc homeostasis, which was closely associated with significant cognitive impairment. It is noteworthy that neutralizing the free zinc released into the synaptic cleft with CaEDTA alleviated sevoflurane-induced cognitive deficits. This finding is consistent with previous studies showing that the chelation of extracellular synaptic zinc can mitigate the adverse effects caused by vesicular zinc dyshomeostasis, even though CaEDTA does not directly affect the content of vesicular zinc [26, 27]. These results underscore the pivotal role of synaptic zinc dysregulation in sevoflurane-induced cognitive dysfunction.

Secondly, this study highlights the potential mechanism by which estrogen regulates synaptic zinc homeostasis and improves cognitive function. Previous research has shown that estrogen plays a critical role in neuronal survival, synaptic plasticity, and anti-inflammatory and antioxidant processes through the activation of various pathways [28, 29]. Our findings suggest that estrogen may modulate the expression of Ap3δ to regulate the zinc transporter protein Znt3, thereby restoring the dynamic balance of synaptic zinc. This mechanism aligns with prior studies on estrogen’s role in improving synaptic zinc homeostasis [23]. Notably, we observed that estrogen supplementation significantly alleviated sevoflurane-induced cognitive impairment, indicating that restoring zinc homeostasis may be a key pathway for its neuroprotective effects on cognitive function.

Similar to previous studies, we also found that the imbalance of synaptic zinc led to increased Tau phosphorylation (AT8), a decrease in neuronal firing frequency, and neuronal damage [30, 31]. Neutralizing the excess free zinc in the synaptic cleft with CaEDTA significantly reduced Tau phosphorylation (AT8), a decrease in neuronal firing frequency, and neuronal damage. Similarly, estrogen treatment decreased synaptic zinc levels by downregulating Znt3 expression, thereby reducing Tau phosphorylation (AT8), a decrease in neuronal firing frequency, and neuronal damage [29]. These findings further confirm the detrimental effects caused by synaptic zinc imbalance. Previous studies have shown that sevoflurane can induce Tau hyperphosphorylation by upregulating TTBK1 expression or enhancing the interaction between Tau and GSK3β [5, 21]. Although our findings suggest that synaptic zinc homeostasis is also involved in modulating Tau phosphorylation, the direct relationship between zinc dysregulation and these kinase-mediated pathways remains unclear and warrants further investigation.

Finally, we found that sevoflurane exposure led to a reduction in the number of synaptic vesicles in the hippocampus, consistent with previous studies [32]. Interestingly, although the number of synaptic vesicles decreased, the content of synaptic zinc increased. This suggests that the content of synaptic zinc may be primarily associated with the zinc transporter Znt3, rather than the number of synaptic vesicles. The underlying mechanisms of this process require further investigation in future studies.

Consistent with previous studies, a temporal separation between molecular analysis and behavioral evaluation in this study highlights the lasting impact of early sevoflurane-induced molecular changes on cognitive function. These effects may be mediated by sustained activation of pathological processes such as Tau aggregation, synaptic disruption, neuroinflammation, and oxidative stress, ultimately contributing to irreversible cognitive impairment [21, 33, 34]. Clarifying these mechanisms will be a key focus of our future work.

However, this study has several limitations. First, although it primarily focuses on the effects of sevoflurane on synaptic zinc homeostasis, other potential pathological mechanisms—such as the release of inflammatory cytokines and neurotransmitter imbalances—may also contribute to sevoflurane-induced cognitive dysfunction and warrant further investigation. Second, we did not directly measure changes in zinc concentration within the synaptic cleft. In addition, since these findings were obtained from aged female mice, it remains unclear whether estrogen would have similar effects in male mice, although existing literature has reported that estrogen also exerts protective effects in males [35]. Finally, our experiments were conducted exclusively in animal models; thus, the safety, timing, and dosage of estrogen administration in clinical practice require further investigation.

In conclusion, this study elucidates the mechanism by which sevoflurane disrupts synaptic zinc homeostasis, leading to cognitive dysfunction, and provides the first evidence that estrogen supplementation can mitigate sevoflurane-induced cognitive impairment by restoring zinc homeostasis. These findings not only enhance the understanding of the pathophysiology underlying anesthesia-related cognitive dysfunction but also offer a solid theoretical foundation and direction for developing perioperative neuroprotective strategies.

## Supplementary Information

Supplementary Material 1. Fig. 1. (A) Open field test for evaluating locomotor activity in mice (*n* = 10/group). (B) Estrogen supplementation increases hippocampal estrogen levels (*n* = 6/group). (C) AAV-Znt3 increases Znt3 protein expression in the mouse hippocampus (*n* = 6/group). Student’s t-test: (A), (B) and (C). *P < 0.05; ***P < 0.001; ns, no significance.

## Data Availability

No datasets were generated or analysed during the current study.
