# Supplementary material for: Estrogen improves sevoflurane-induced cognitive dysfunction by regulating synaptic zinc homeostasis
Source: Mol Med. 2025 Oct 14;31:312. doi: 10.1186/s10020-025-01364-6 (PMC12522931; doi:10.1186/s10020-025-01364-6)
Supplement: Supplementary file 1 — Supplementary Material 1. Fig. 1. (A) Open field test for evaluating locomotor activity in mice (n = 10/group). (B) Estrogen supplementation increases hippocampal estrogen levels (n = 6/group). (C) AAV-Znt3 increases Znt3 protein expression in the mouse hippocampus (n = 6/group). Student’s t-test: (A), (B) and (C). *P < 0.05; ***P < 0.001; ns, no significance. [file 10020_2025_1364_MOESM1_ESM.docx]

**Supplementary Figure 1**

**
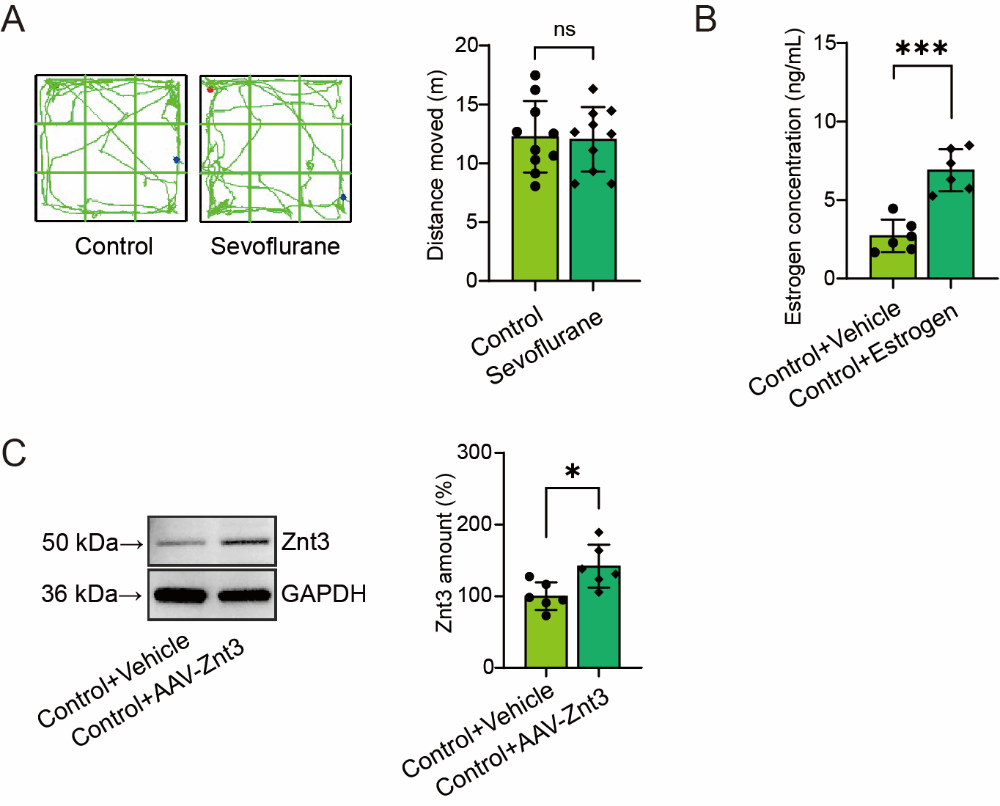
**

**Supplementary Figure 1** (A) Open field test for evaluating locomotor activity in mice (n = 10/group). (B) Estrogen supplementation increases hippocampal estrogen levels (n = 6/group). (C) AAV-Znt3 increases Znt3 protein expression in the mouse hippocampus (n = 6/group). Student’s t-test: (A), (B) and (C). **P*<0.05; ****P*<0.001; ns, no significance.
